# Sinus Augmentation with Simultaneous, Non-Submerged, Implant Placement Using a Minimally Invasive Hydraulic Technique

**DOI:** 10.3390/medicina56020075

**Published:** 2020-02-13

**Authors:** Liat Chaushu, Gavriel Chaushu, Hadar Better, Sarit Naishlos, Roni Kolerman, Juan Manuel Aragoneses, José Luis Calvo-Guirado, Joseph Nissan

**Affiliations:** 1 Department of Periodontology and Implant Surgery, The Maurice and Gabriela Goldschleger School of Dental Medicine, Tel Aviv University, Tel Aviv 6997801, Israel; kolerman@netvision.net.il; 2 Department of Oral and Maxillofacial Surgery, The Maurice and Gabriela Goldschleger School of Dental Medicine, Tel Aviv University, Tel Aviv 6997801, Israel; gabi.chaushu@gmail.com; 3 Department of Oral and Maxillofacial Surgery, Rabin Medical Center, Petah Tiqwa, 4922297 Israel; nissandr@gmail.com; 4 Private practice in Oral and Maxillofacial Surgery, Tel Aviv 6997801, Israel; bettermed@gmail.com; 5 Department of Pedodontics, The Maurice and Gabriela Goldschleger School of Dental Medicine, Tel Aviv University, Tel Aviv 6997801, Israel; river554@gmail.com; 6 Department of Dental Research in Universidad Federico Henriquez y Carvajal, Santo Domingo 10107, Dominican Republic; jmaragoneses@gmail.com; 7 Department of Oral and Implant Surgery, Faculty of Health Sciences, Universidad Católica de Murcia, Murcia 30107, Spain; jlcalvo@ucam.edu; 8 Department of Oral Rehabilitation, The Maurice and Gabriela Goldschleger School of Dental Medicine, Tel Aviv University, Tel Aviv 6997801, Israel

**Keywords:** sinus lift, Maxillent dental implant, sinus elevation, membrane elevation

## Abstract

*Background and objectives*: To evaluate whether sinus augmentation, using a minimally invasive implant device, via a non-submerged surgical approach, might negatively influence the outcome. *Materials and Methods:* A retrospective cohort study was conducted by evaluating patients’ files, classifying them into two groups. Fifty patients (22 men 28 women) were included in the study, 25 in each group. The use of an implant device based on residual alveolar ridge height for sinus augmentation, radiographic evaluation, insertion torque, membrane perforation, post-operative healing, and a minimum of 12 months follow-up were evaluated. *Results:* The mean residual alveolar ridge height was 5.4 mm for the non-submerged group and 4.2 mm for the submerged group. There were no intraoperative or postoperative complications (including membrane perforations). The mean insertion torque was 45 N/cm for the study group and 20 N/cm for the control group. Complete soft tissue healing was observed within three weeks. Mean bone gain height was 8 mm for the study and 9.3 mm for the control group. All implants osseointegrated after 6–9 months of healing time. Mean follow-up was 17.5 months, range 12–36 months. Marginal bone loss at last follow-up was not statistically significantly different: 1 mm in the non-submerged vs. 1.2 mm in the submerged group. *Conclusions:* Submerged and non-submerged healing following maxillary sinus augmentation was comparable provided residual alveolar ridge height >5 mm and insertion torque >25 N/cm.

## 1. Introduction

The first rule of medicine is "primum non nocere" [1]. Surgery is heading towards minimally invasive intervention resulting in minimal postoperative morbidity, loss of working days, improved healing, and reduced operation time being the main benefits [2,3,4]. Non-submerged implant placement presents a viable alternative to staged placement in implant dentistry [5,6]. The prerequisites for such a procedure include sufficient uncompromised bone dimensions in the implantation site and ample initial insertion torque enabling the primary stability of the inserted implant [5,6]. The principal risks of the technique are a loss of implant stability leading to inflammation and early implant failure [7]. In the posterior maxilla cases, oroantral fistula and poor esthetical results might be additional unfortunate outcomes [7]. Implant placement in the posterior maxilla is often challenging due to limited bone volume availability [8,9,10,11,12,13,14,15,16,17,18,19,20,21,22,23,24,25,26]. Longer and broader implants may have a biomechanical advantage, leading to long-term survival [27]. Recently, a minimally invasive implant device iRaise, Maxillent, Herzliya, Israel, was introduced [11,12]. The implant length of this device is based on the residual alveolar ridge height (RAH; 13 mm implant length—3–5 mm RAH; 14.5 mm implant length—5–6.5 mm RAH; and 16 mm implant length—6.5–8 mm RAH) [11,12]. A non-submerged approach combined with an implant-device based on residual alveolar ridge height has not been described yet. The null hypothesis of the present study was that sinus augmentation using a residual alveolar ridge height-based implant device via a non-submerged surgical approach yields results similar to those obtained via a submerged one. Primary outcome parameters included short term implant survival, bone gain, and marginal bone loss at last follow-up. Whereas membrane perforation, postoperative infection, and soft tissue healing were the secondary outcomes.

## 2. Materials and Methods 

The study was approved by the ethical committees of the Israeli Ministry of Health, (HTA5284, date of approval 24 September 2017) and Tel Aviv University. A retrospective cohort study was conducted by evaluating patients’ files, classifying them into two groups. Power analysis was conducted to estimate the number of cases needed, assuming a level of significance 5%, desired power of 80%, identical allocation ratio for both groups, and estimated effect size of 0.5 standard deviations. Results showed that the sample size required is 50 cases, equally distributed for both groups.

The records of the first fifty (25 studies and 25 control) adults (22 men 28 women) compatible with the inclusion and exclusion criteria were included. Age ranged 45–70 years (mean age 54 ± 3 for the control and 48 ± 2 for the study group). Inclusion criteria—a moderately to severely atrophic posterior maxilla with residual alveolar bone ≥4 mm (in the location intended for non-submerged implant placement) as demonstrated by a preoperative computerized tomography (CT) imaging, no systemic or local disorders that could affect sinus augmentation surgery, a minimally invasive sinus lift operation using an implant device, adequate pre and postoperative records—radiographic preoperative (CT) and post-operative records (CT or panoramic, periapical); records of insertion torque, membrane perforation, post-operative healing; a minimum 12 months follow-up; and periapical radiographs at last follow-up. 

The exclusion criteria were chronic steroid therapy, uncontrolled diabetes, cardiovascular disease prohibiting extensive surgery, past head and neck radiation therapy, maxillary sinus cysts, active chronic sinusitis, smoking more than ten cigarettes per day, and the inability to perform proper oral hygiene. Only patients treated by one oral surgeon (GC) between 2008 and 2017 were included. The standard surgical protocol developed is further described in the material and methods section. The records were searched starting 2008. Initially, only a submerged technique was used. The first 25 patients compatible with the inclusion criteria served as control. During 2013 the ability to perform hydraulic sinus elevation in a non-submerged technique was presented in an international congress by the Austrian group. From 2014 the non-submerged technique was initiated by one of the authors as a clinical practice. The first 25 patients compatible with the non-submerged technique and study criteria were included in the present study that was initiated in 2017. 

In the initial diagnostic meeting, alternative treatment plans, such as removable prostheses and bridges when applicable, were also discussed in detail with each patient. Those choosing sinus augmentation using the hydraulic technique were included. The first outcome parameters included membrane perforation, implant survival, bone gain, and marginal bone loss at last follow-up. The device used for sinus augmentation was a self-tapping endosseous dental implant (iRaise^™^, Maxillent, Herzliya, Israel) [11,12]. It contains an internal channel that allows the introduction of liquids through the implant body and into the maxillary sinus. 

### 2.1. Standard Surgical Procedure

**Figure 1:**
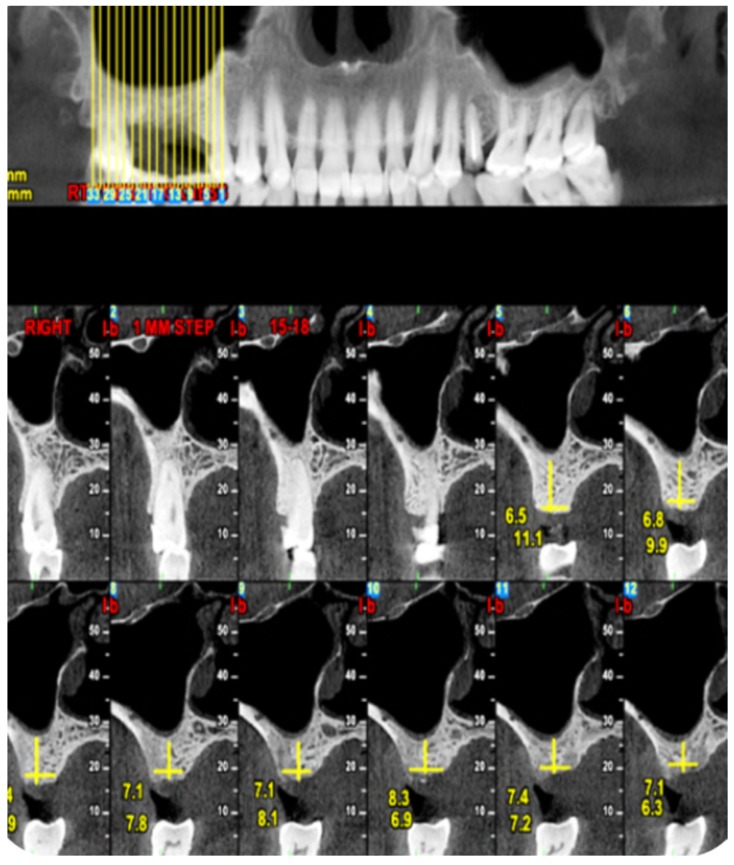
Preoperative CT view demonstrating residual alveolar ridge height.

The diagnostic CT was reviewed by two of the investigators who were not the surgeon (LC and JN) and was double-checked. Preoperative imaging demonstrating at least 3 mm residual alveolar ridge height was a prerequisite (Figure 1).

**Figure 2:**
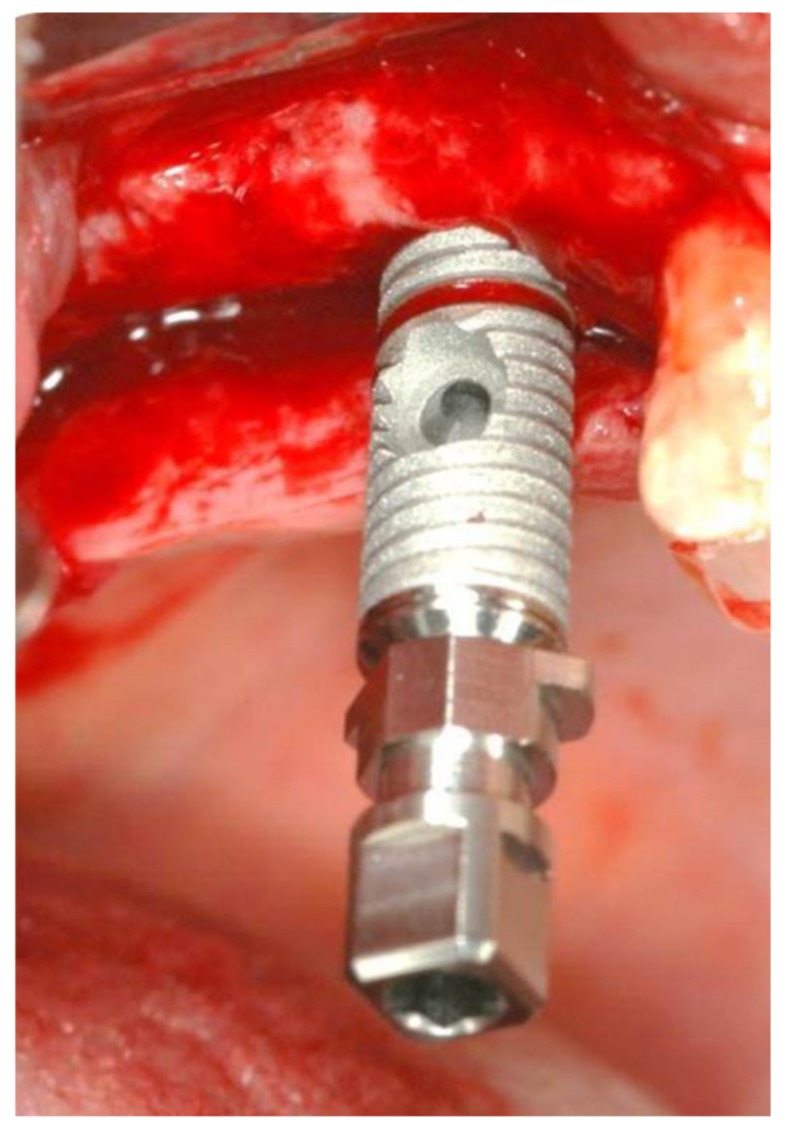
iRaise implant insertion.

Prophylactic antibiotics were administered (1 g of amoxicillin, 1 hour before the procedure). The patient performed a mouth wash for 1 minute with chlorhexidine gluconate 0.2% solution before surgery. Surgery commenced with local anesthesia and a crestal incision, without vertical extensions, along the maxillary ridge. Relatively small full-thickness mucoperiosteal flaps were reflected buccally and lingually. The osteotomy site was marked with a small round bur. An osteotomy was started at the implantation site with a 2.8 mm drill to a depth of 3 mm using a stopper. A periapical radiograph with a depth guide was performed in order to verify the drilling angulation and depth. The osteotomy site was widened with two diameters under drilling of to the desired width (3.2 mm for 4.2 mm; 3.65 mm for 5 mm) according to fabricant (iRaise^™^, Maxillent, Herzliya, Israel) sequence. The sinus floor was weakened/opened using a drill with an active diamond tip designed to atraumatically penetrate the sinus floor under the Schneiderian membrane without damaging the membrane. The length of the implant (ranging from 13 to 16 mm; mean 14 ± 085 mm) was selected based on the residual bone height: a 13-mm length implant was used for bone heights of up to 5  mm, a 14.5-mm length implant was used for bone heights of up to 6.5 mm, and a 16-mm length implant was used for bone heights of up to 8 mm. The implant was first inserted into the osteotomy until it reached the end of the prepared osteotomy and slowly advanced until the sinus floor was penetrated (<1 mm; Figure 2).

**Figure 3:**
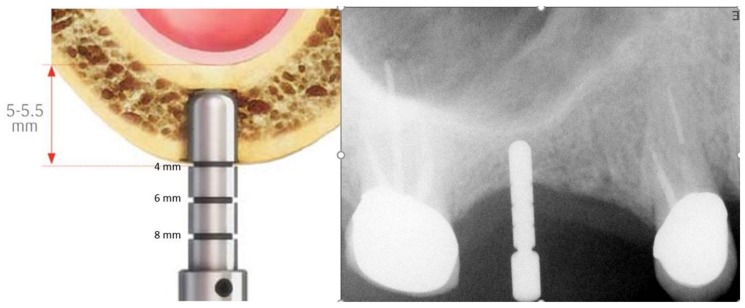
Drilling height verification.

A periapical radiograph was performed with a film holder (Dentsply Rinn, Elgin Rinn Corporation, XCP, PA, USA) in some cases in order to determine whether the implant penetrated the sinus floor (Figure 3). 

**Figure 4:**
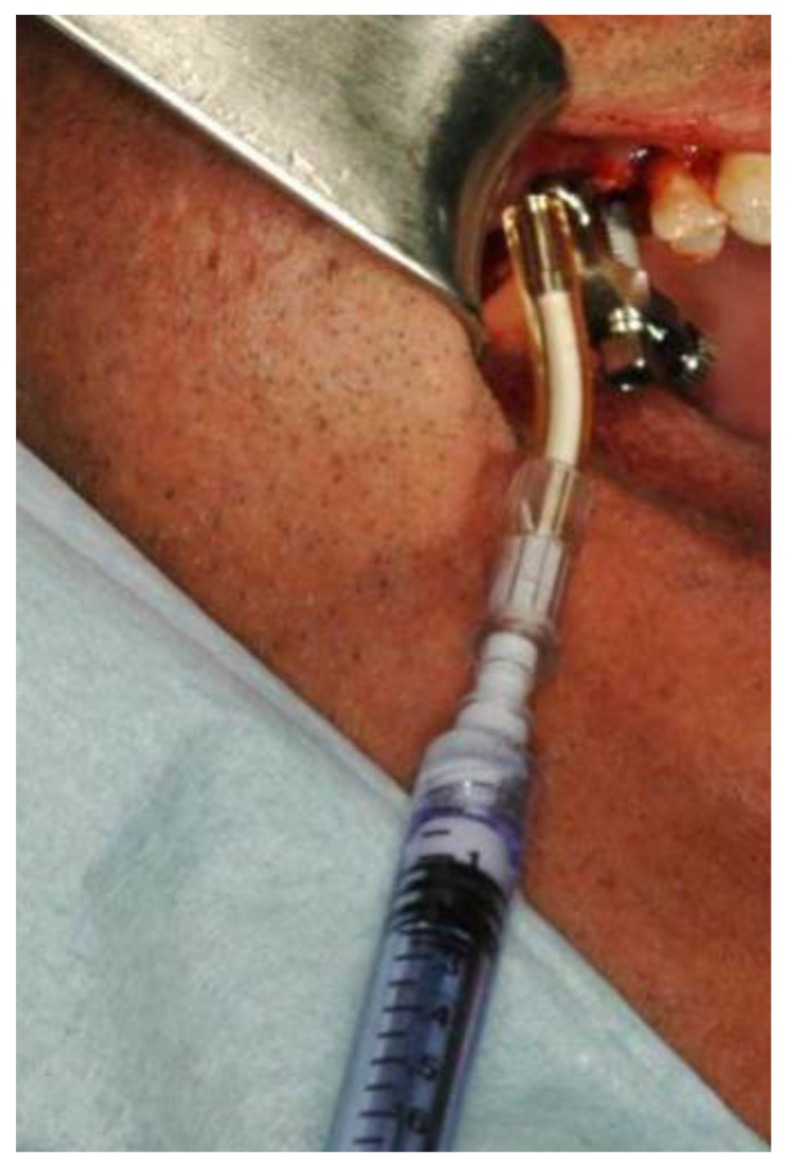
Bone graft coming out of the second implant osteotomy.

A saline syringe (0.9% sodium chloride sterile saline solution) was connected to the implant via the tubing port. Saline solution was gently injected through the implant and into the sinus. Slight bleeding was noted in the retracted saline solution. Typically, 2 cc of saline was required. The saline solution was retracted back into the syringe, and the saline syringe was disconnected from the tubing port. A flowable bone graft filled syringe was then connected to the tubing port. The bone graft material was then slowly injected through the implant into the sinus (Figure 4). 

**Figure 5:**
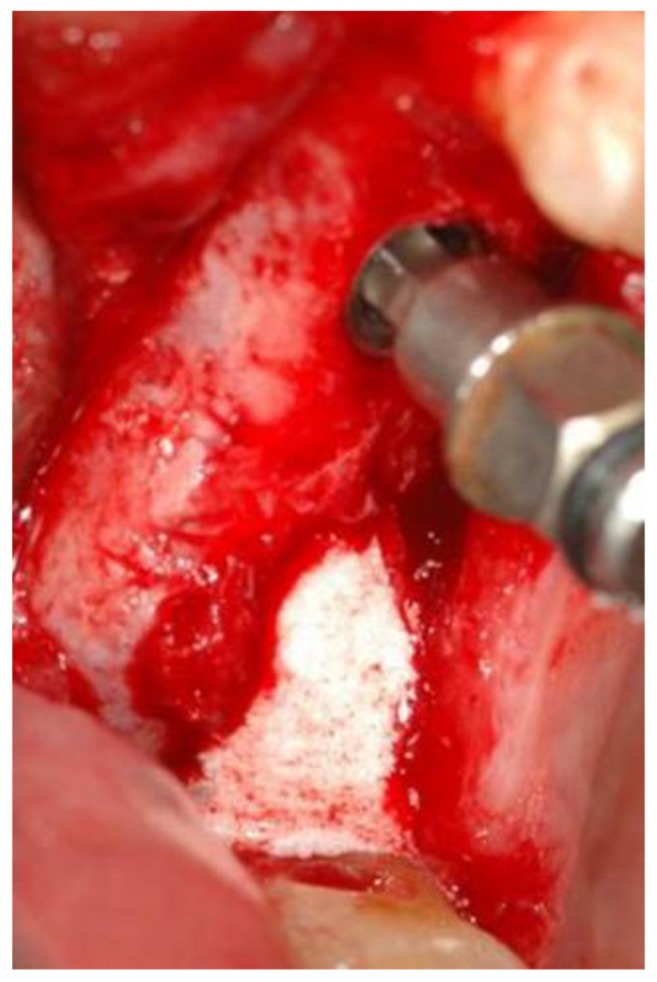
Bone graft injected.

The bone graft syringe and the tubing port were disconnected from the implant. Two injectable bone grafting materials were used at the discretion of the operator, a synthetic injectable gel comprised of granules of biphasic calcium phosphate in the suspension of a soluble polymer or β tricalcium phosphate granulate suspended in a hyaluronic acid matrix. The implant was then fully inserted through the osteotomy until the coronal aspect of the implant was aligned with the maxillary alveolar crest. Bone graft coming out of the second implant osteotomy reassured the intact Schneiderian membrane (Figure 5).

**Figure 6:**
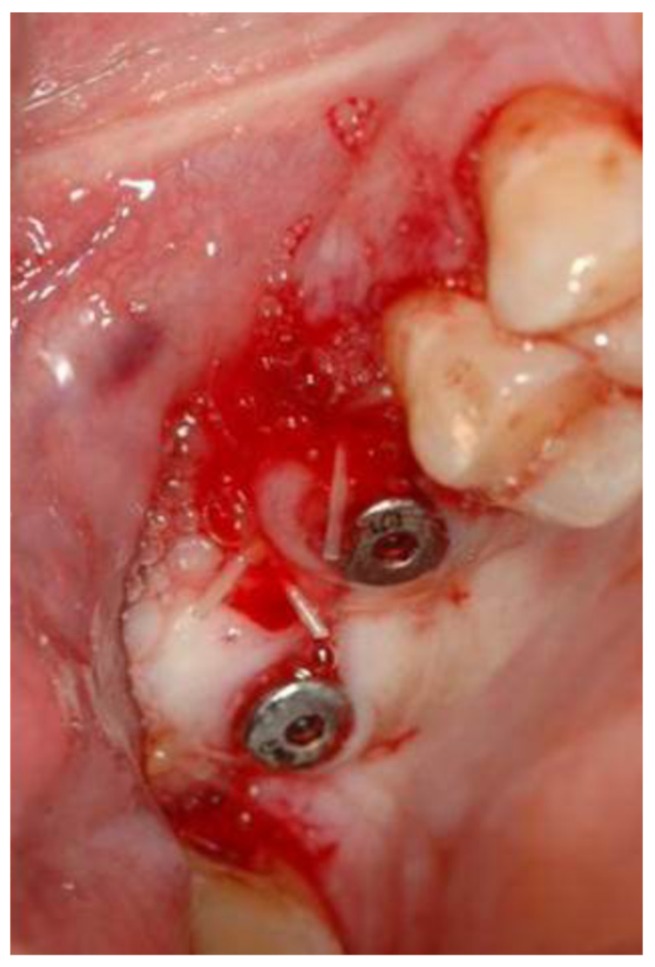
Primary closure with healing caps in place.

Additional implants were placed. Insertion torque of all implants was recorded. Cover screws were assembled in the submerged group, and healing caps were assembled in the non-submerged group. The gingival flaps were then sutured by polyglactin 910 monofilament braided 3-0 (Vicryl Rapide™, J&J Ethicon, Minnesota, USA; Figure 6). A final periapical radiograph was used for baseline documentation of both implants and bone graft.

Following the procedure, the patients were instructed to use mouth rinsing for 1 minute with 0.2% chlorhexidine solution, twice a day, for ten days. Postoperative analgesia was used as needed. Nose drops (topical decongestants such as oxymetazoline) were used in the relevant nostril twice a day for a week. Antibiotics were prescribed at the clinician’s discretion (as usually given in bone grafting procedures): Amoxicillin 500 mg ×  3  for seven days. Second stage surgery was performed after 6–9 months for the non-submerged group.

### 2.2. Marginal Bone Loss

A periapical radiograph was performed with a film holder (Dentsply Rinn, Elgin Rinn Corporation, XCP, PA, USA). Measurements of crestal bone loss at the mesial and distal parts of the implant were made by computer analysis using periapical radiographs, similar to the method of measurement described by Turkyilmaz et al. [28]. Periapical radiographs were taken after implantation was compared to radiographs at the follow-up appointment using Image-J (Image Processing Software) [29,30]. The length of the implant was used to cancel the X-ray distortion. Crestal bone level was measured as the distance from the implant-abutment junction to the adjacent crestal bone (mesial and distal). The differences between the measurement at implant placement vs. follow-up appointment were defined as crestal bone loss.

### 2.3. Statistical Analysis

All data were taken from the patients’ files and processed statistically using SPSS version 25. To compare potential confounders (age and initial height of implant) between groups in continuous and categorical measures, Mann–Whitney and chi-square tests were conducted respectively. To test main outcomes between groups after adjusting for confounders, the Analysis of Co-Variance procedure (ANCOVA) was conducted. Finally, Spearman correlations were used to assess the correlation between non-submerging and bone loss, insertion torque and bone loss, residual alveolar ridge height and bone loss, age, and bone loss. The differences were considered significant if the *p* value was less than 0.05.

## 3. Results

**Figure 7:**
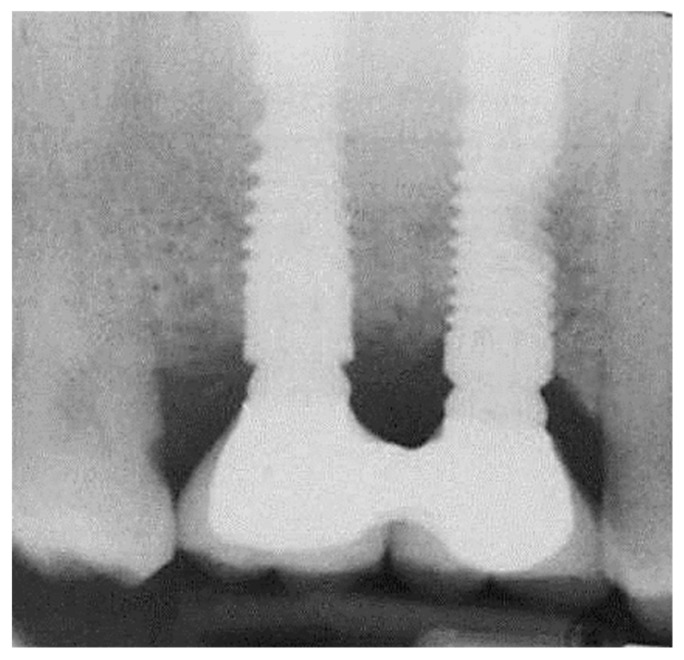
Periapical radiograph at the 3-year follow-up.

Fifty patients (22 men and 28 women) were included in the study, 25 in control, and 25 in the study group. Groups had a similar proportion of males (56%) and females (44%). However, groups were different in age and the initial height of implants. The mean age was in the 54 ± 3 range for the control and 48 ± 2 for the study group (*p* < 0.01). The mean initial height was 4.21 ± 0.5 for the control and 5.44 ± 0.76 for the study group (*p* < 0.01). Therefore, age and initial height were statistically controlled as confounders. A total of 25 iRaise (Maxillent, Herzliya, Israel) implants were used for both the study and control group. Implant length ranged 13–16 mm, and the diameter was 4.2 or 5. Additional implants placed were 13 mm long with a diameter of 3.75 or 4.2 mm. Mean residual alveolar ridge height was 5.4 mm (range 5–7 mm) for the study group and 4.2 mm (range 4–6 mm) for the control group. Mean insertion torque was 44.40 ± 9.57 (range 25–55) N/cm for the study group and 20.40 ± 6.27 (range 10–25) N/cm for the control group (*p* < 0.01). There were no intraoperative or postoperative complications. Complete soft tissue healing was observed within three weeks. There were no intraoperative difficulties in introducing fluids. No postoperative infections were recorded. The mean one gain height for the study group was 7.80 ± 0.5 mm and 9.3 ± 0.5 mm for the control group (*p* < 0.01). Second stage surgery was performed after 6–9 months of healing time only in the submerged group. All implants were osseointegrated at second-stage surgery. All implants were restored with the fixed implant-supported prosthesis. Mean follow-up was 17.5 months, range 12–36 months. Comparing marginal bone loss at last follow-up after controlling age and initial height, showed no statistical difference: 1 ± 0.4 mm in the non-submerged vs. 1.2 ± 0.5 mm in the submerged group (*p* = 0.658; Figure 7). No significant correlations were found between bone loss and non-submerging; insertion torque; residual alveolar ridge height; and age. 

## 4. Discussion

Expectations and preferences of patients seeking dental implants today are high. When asked, they mention treatment predictability and fixed implant-supported prostheses as their main demands. They are willing to shorten treatment time, yet without increasing failure rates and postoperative morbidity. Patients are ready to pay the additional costs associated with computed tomography and new surgical techniques, especially those offering treatment alternatives with fewer interventions [31]. A recent systematic review, meta-analysis and sequential trial analysis of submerged versus non-submerged implant healing used implant failure as well as marginal bone level changes between submerged and non-submerged healed dental implants as the primary outcome parameters [6]. Similarly, those two outcome parameters were used in the present study for the evaluation of non-submerging. Studies concerning transcrestal sinus augmentation considered intrasinus bone gain as a major outcome parameter [32]. Hence, the present study, evaluating transcrestal hydraulic sinus augmentation motivated us to use intrasinus bone gain as the third significant outcome parameter. One of the limitations of the present study is the sample size. However, most of the studies concerning outcome of no difference between submerged and non-submerged placement are of a similar magnitude and similar patient number (Torzkaban et al. 24 test vs. 24 control; Nemli et al. 19 vs. 19; Gulati et al. 16; Tallarico et al. 29 vs. 18; and Enkling et al. 22 vs. 22) [33,34,35,36,37]. The high survival rates motivated us to add two additional parameters, bone loss and new bone gain, to further support the lack of difference between the two surgical approaches. Since data for this study were obtained retrospectively, data only for age and sex were gathered as potential confounders. The present study supports the use of a minimally invasive implant device in a non-submerged technique for maxillary sinus augmentation with simultaneous implant placement. This hydraulic technique for sinus augmentation was extensively described in the literature [38,39,40,41,42,43,44,45,46,47]. Chen et al. reported in 2009 a ten-year follow-up. The operators were able to predictably elevate the Schneiderian membrane, introduce bone graft, and place implants [38,39,40,41,42,43,44,45,46,47].

The hydraulic technique improves two major concerns compared to the other transcrestal techniques. First, Schneiderian membrane perforation rate and second, new bone gain. Kher et al. [48] reported no sinus membrane perforations and mean gain in bone height post-operatively of 10.31 ± 2.46 mm. Bensaha et al. [43] reported also no membrane perforations and mean bone gain of 12.03 ± 2.1 mm. Tallarico et al. [49] reported no intraoperative or postoperative adverse events as membrane tears. The mean bone gain was 10.9 ± 2.43 mm (range 5.3–16.5). Another recent study by Gatti et al. [50] also reported no membrane tears.

The present study further contributes by describing the ability to assemble healing caps in a single intervention. This way, more patients may be willing to undergo sinus augmentation procedures and overcoming their fears of multiple interventions. The results of the present study are compatible with previous studies reporting the outcome of non-submerged implant placement [5,6]. The non-submerged crestal sinus floor elevation with simultaneous implant placement in the present study is reported as safe, effective, decreasing surgical discomfort, and trauma. The minimum insertion torque was 25 N/cm for the study group. Therefore, we need to limit the non-submerged indication only when similar stability is achieved. The possibility of non-submerge implants with lower insertion torque awaits future evaluation. The effect of non-submerged implant surgery on soft tissue was previously assessed. The results suggest that non-submerged implant surgery provides esthetic and favorable soft tissue results [5,6]. Similar results were also observed in the current study (a complete soft tissue healing was noted within three weeks), allowing optimal soft tissue manipulation during prosthetic restoration. Marginal bone loss at last follow-up was not statistically significant in the non-submerged vs. the submerged group. This criterion demonstrated that the initial presence of healing caps did not result in an overload of the implants leading to more marginal bone loss rendering non-submerged implants to an increased chance for peri-implant disease. The present study demonstrated that the use of a non-submerged approach yields fruitful results; however, it is recommended only for those practitioners with advanced clinical experience, especially those using the device successfully in a submerged approach. The non-submerged approach, together with the minimally invasive implant device used in this study, reduced postoperative patient morbidity, decreased surgical time, and promoted soft tissue healing.

## 5. Conclusions

The null hypothesis of the present study, sinus augmentation using a residual alveolar ridge height-based implant device via a non-submerged surgical approach yield results similar to those obtained via a submerged one, was approved. Submerged and not submerged healing following maxillary sinus augmentation was comparable, provided residual alveolar ridge height >5 mm and insertion torque >25 N/cm.
